# Immune regulation and prognosis indicating ability of a newly constructed multi-genes containing signature in clear cell renal cell carcinoma

**DOI:** 10.1186/s12885-023-11150-4

**Published:** 2023-07-12

**Authors:** Ziwei Gui, Juan Du, Nan Wu, Ningning Shen, Zhiqing Yang, Huijun Yang, Xuzhi Wang, Na Zhao, Zixin Zeng, Rong Wei, Wenxia Ma, Chen Wang

**Affiliations:** 1 Department of Pathology, Second Clinical Medical College of ShanXi Medical University, Tai Yuan City, ShanXi Province China; 2 Department of Anesthesiology, Second Hospital of ShanXi Medical University, Tai Yuan, ShanXi Province China; 3 Department of Pathology, Second Hospital of ShanXi Medical University, No.382 Wuyi Road, Tai Yuan, ShanXi Province 030000, China

**Keywords:** Clear cell renal cell carcinoma (ccRCC), Immune response, LASSO analysis, Gene signature, Prediction biomarker

## Abstract

**Background:**

Clear cell renal cell carcinoma (ccRCC) is the most common renal malignancy, although newly developing targeted therapy and immunotherapy have been showing promising effects in clinical treatment, the effective biomarkers for immune response prediction are still lacking. The study is to construct a gene signature according to ccRCC immune cells infiltration landscape, thus aiding clinical prediction of patients response to immunotherapy.

**Methods:**

Firstly, ccRCC transcriptome expression profiles from Gene Expression Omnibus (GEO) database as well as immune related genes information from IMMPORT database were combine applied to identify the differently expressed meanwhile immune related candidate genes in ccRCC comparing to normal control samples. Then, based on protein–protein interaction network (PPI) and following module analysis of the candidate genes, a hub gene cluster was further identified for survival analysis. Further, LASSO analysis was applied to construct a signature which was in succession assessed with Kaplan–Meier survival, Cox regression and ROC curve analysis. Moreover, ccRCC patients were divided as high and low-risk groups based on the gene signature followed by the difference estimation of immune treatment response and exploration of related immune cells infiltration by TIDE and Cibersort analysis respectively among the two groups of patients.

**Results:**

Based on GEO and IMMPORT databases, a total of 269 differently expressed meanwhile immune related genes in ccRCC were identified, further PPI network and module analysis of the 269 genes highlighted a 46 genes cluster. Next step, Kaplan–Meier and Cox regression analysis of the 46 genes identified 4 genes that were supported to be independent prognosis indicators, and a gene signature was constructed based on the 4 genes. Furthermore, after assessing its prognosis indicating ability by both Kaplan–Meier and Cox regression analysis, immune relation of the signature was evaluated including its association with environment immune score, Immune checkpoint inhibitors expression as well as immune cells infiltration. Together, immune predicting ability of the signature was preliminary explored.

**Conclusions:**

Based on ccRCC genes expression profiles and multiple bioinformatic analysis, a 4 genes containing signature was constructed and the immune regulation of the signature was preliminary explored. Although more detailed experiments and clinical trials are needed before potential clinical use of the signature, the results shall provide meaningful insight into further ccRCC immune researches.

**Supplementary Information:**

The online version contains supplementary material available at 10.1186/s12885-023-11150-4.

## Background

Renal cell carcinoma has been the most common kidney malignancy which comprises ccRCC and non clear cell Renal Cell Carcinoma (nccRCC), and ccRCC accounts for approximately 70% ~ 75% of all the cases [1, 2]. Attributing to the rapid development of molecular pathology, the genome mechanism behind ccRCC occurence has been gradual clear, of which short arm of chromosome 3 (3p) genes variations were showing defining characteristic roles involving most importantly VHL gene known by symbolic “double hit”. The aberrant change of VHL including gene mutation and promoter methylation causes the “first hit”, followed by "second hit", namely the 3p chromosome deletion leading to tumor occurrence [3–5]. Besides VHL, other 3p gene variations were also reported in ccRCC, for instance SETD2 [6], BAP1 [7] and PBRM1 [8], which were reported to be survival related.

Regarding the clinical treatment, over the past two decades, molecular targeted therapies and immune therapies have been showing great potential. Currently, at least 13 drugs in 6 categories have been approved for metastatic ccRCC, including VEGFR, mTORC1, c-Met, FGFR inhibition, cytokines, and most recently anti PD-1/PD-L1 immune checkpoint inhibitors (ICIs) which has been a promising pillar of nowadays clinical treatment [4, 9]. Multi clinical trials most notably KEYNOTE-564 results supported ccRCC as immune sensitive and demonstrated the efficiency of ICIs in advanced patients clinical treatment using independent ICI therapies or ICI + TKI combination therapies [10].

Although evidence-based medicine supported RCC as one of the malignancies that could benefit from neo-adjuvant PD-1/PD-L1 blockade [11–13], the immune response obviously vary among individuals indicating the importance of usable and effective immune biomarkers for selecting the potential patients that were most likely to be able to benefit from the treatment [14]. Currently, PD-L1 expression, MSI and tumor mutation burden (TMB) were three most widely used biomarkers in other types of cancers, however, their use in ccRCC are still in dispute.

As for PD-L1 expression, although it has been an effective biomarker in other cancers for predicting immune response [15–17], it’s function in ccRCC is inconclusive yet. For example, clinical trial Checkmate025 showed that advanced ccRCC patients benefited from immunotherapy regardless of PD-L1 expression [18, 19]. Checkmate214 revealed that the objective remission rate (ORR) in immunotherapy received group was higher than that in sunitinib treatment group no matter PD-L1 expression was higher or less than 1% [20, 21]. Meanwhile, Keynote426 results also revealed that both PD-L1 negative and positive ccRCC patients benefited from pablizumab and axitinib combination therapy [22, 23].

Meanwhile, as for the use of TMB in renal malignancy, Robert M Samstein.et al. reported an pan-cancer MSK-IMPACT genome sequencing analysis based on 1600 cases of cancer samples, of which 151 cases were RCC samples, and the results showed that TMB was non significant related with ccRCC overall survival [24]. And since microsatellite instability (MSI-H and dMMR) is very rare in RCC patients, MSI is not supported by evidence-based medicine yet to be an effective immune biomarker [14, 25].

Above all, ICIs has been an promising treatment in clinical ccRCC, but the effective biomarkers for immune response prediction are still lacking, it is of great importance to keep exploring ccRCC genome and identifying new potential immune response biomarkers thus aiding more precise understanding of the disease and shedding promising light on further clinical immunotherapy application.

In the study, multi online ccRCC transcriptome profiles, our local hospital patients samples as well as various bioinformatic analysis tools were combine used to explore ccRCC genome data, identifying the survival related meanwhile immune regulation associated genes and constructing a potential immune signature, further necessary signaling mechanism was preliminary explored. The results shall provide meaningful insights to the unearth of potential new immune biomarkers and shed promising light on further ccRCC immune researches.

## Materials and methods

### Data source: ccRCC transcriptome data from GEO database

From GEO online database, we widely screened ccRCC related profiles for exploring the changed genome information in cancer comparing to normal renal samples. The selection criteria of GEO profiles were set as: 1. the information of profiles were based on human tissues (not animal models of any species); 2. the samples type was solid tissue (not tumor cell lines); 3. the containing data were mRNA/ cDNA/ transcriptome sequencing data; 4. covering both ccRCC cancer and normal renal control samples; 5. each profile should contain at least 40 or more samples.

Based on above selection criteria, a total of five ccRCC cDNA expression profiles were selected, including four profiles namely GSE53000 [26], GSE53757 [27], GSE68417 [28] and GSE71963 [29] containing a total of 186 cases of ccRCC samples and 108 normal kidney samples were selected for further potential genes selection. Meanwhile, another datasets GSE22541 which includes 68 cases of ccRCC samples was applied as validating data source. (Table S1 for detailed information of the profiles including samples amount, contributors and accessed online website).

### Data processing: identify the differently expressed meanwhile immune related genes in ccRCC comparing to normal control

The GEO transcriptome data were used to 1. explore the differently expressed genes in ccRCC comparing to normal kidney samples; 2. combine with IMMPORT immune database [30] for collaborate identify the differently expressed meanwhile immune regulation related genes.

To reveal the aberrant differently expressed genes in ccRCC comparing to normal control samples, four GEO profiles GSE53000, GSE53757, GSE68417 and GSE71963 were in succession analyzed with GEO2R which was provided pared with each GEO profile. The analysis criteria was set as adjusted *P* value < 0.05 meanwhile |log2FC|< 1, 1 ≤|log2FC|< 2, 2 ≤|log2FC|< 3 and |log2FC|≥ 3 respectively, thus the genes expression change distribution namely the genes that were < twofold, 2 ~ fourfold, 4 ~ eightfold and > eightfold different in cancer versus normal control would be preliminary understood.

Then, Venn diagram [31] would be used to identify the immune related genes from all the high level differently expressed genes based on IMMPORT immune genes list, therefore, the genes that were preliminary supported to be both aberrant changed expressed and immune regulation related were selected as candidate genes for further analysis.

### Protein–protein interaction (PPI) network construction and function module analysis of the candidate genes

After identifying the differently expressed meanwhile immune related candidate genes, STRING [32], which is short for Search Tool for the Retrieval of Interacting Genes was applied to construct the PPI network of above selected genes for observing the interaction between individual genes. Further, based on PPI network, Molecular Complex Detection (MCODE) function of Cytoscape3.6.0 software [33] was used to analyze the promising function modules (gene clusters sharing similar function) from the gene nest.

Further, Gene ontology analysis (GO) and Kyoto Encyclopedia of Genes and Genomes (KEGG) [34] were used to annotate basic biological attributes of the list of genes in each module including their main cellular location, involved biological processes, molecular functions and the signaling pathways they mainly enriched in. The module that was predicted to be most related with tumor immune modulation and possess the highest module score would be highly focused and identified as a potential gene cluster for further analysis.

### Univariate survival combine with Cox regression analysis of immune related gene cluster for hub genes

Following the identification of the immune related gene cluster, each gene in the module would be in succession brought for firstly univariate survival analysis by UALCAN [35] and GEPIA [36], which have been two effective online services for survival analysis. Then, the genes that were supported by both univariate analysis methods to be statistical significantly associated with ccRCC survival would be processed for multivariate COX regression analysis based on TCGA ccRCC data using SPSS19.0 analysis. Any gene that was indicated by all three analysis to be associating with patients survival would be identified as credible prognostic indicating hub genes and processed for next step interpretation.

### Estimation of hub genes’ physicochemical properties

For understanding the basic information of the selected hub genes, ProtParam [37], ProtScale [38], and Human Protein Atlas [39] were combine used. As for the physicochemical properties of genes, ProtParam and ProtScale were applied to understand the basic information of the genes encoding proteins including the aminoacid composition, estimated molecular weight and protein half life, computed protein instability index and theoretical isoelectric point, as well as hydrophobicity and hydrophilicity of proteins.

Besides ProtParam and ProtScale, Human Protein Atlas is also an effective and well used online service for interpreting certain proteins information, in the study, it was applied to predict the cellular location of the selected hub genes for the convenience of further clinical test.

Additionally, UALCAN as well as GEPIA, which have been two resourceful web services constructed based on TCGA and GTEx programs were in succession accessed to observe the expression difference of hub genes in broad-spectrum human cancers comparing to corresponding normal control samples, especially in ccRCC versus normal renal tissues.

### Quantitative real-time PCR (QPCR) experiment for evaluating the expression change of selected hub genes in cancer vs normal tissues

Besides above UALCAN as well as GEPIA online analysis, 30 pairs of ccRCC cancer tissues and adjacent paracancerous normal renal tissues which were all collected from our local hospital were used for validating the expression changes of selected hub genes. All the patients tissues were collected from surgeries at local hospital General Surgery Department and sent for pathology examination then being long-term stored at Pathology Department Biobank. The Informed consent from the patients as well as the approval by the Hospital Institutional Board were both obtained (Second Hospital of ShanXi Medical University, China).

The mRNA of 30 pairs of ccRCC cancer and adjacent normal renal tissues were extracted using RNAiso-Plus (TAKARA, DaLian, China). And then1 μg extracted mRNA was used for cDNA synthesis using cDNA synthesis kit (TAKARA, DaLian, China) following operating instruction. Further, qPCR was performed on Roche Light Cycler z 480 and the primers of the tested hub genes used during the process were listed as below:

MMP9:

Former: AGACCTGGGCAGATTCCAAAC

Reverse: CGGCAAGTCTTCCGAGTAGT

NFKB1:

Former: AGCACGACAACATCTCATT

Reverse: CAGGCACAACTCCTTCAT

IRF7:

Former: CCCACGCTATACCATCTACCT

Reverse: GATGTCGTCATAGAGGCTGTTG

HMOX1:

Former: TGCCAGTGCCACCAAGTTCAAG

Reverse: TGTTGAGCAGGAACGCAGTCTTG

GAPDH:

Former: AGAAGGCTGGGGCTCATTTG

Reverse: AGGGGCCATCCACAGTCTTC

The PCR cycling condition was set as: 95 °C 10 min for 1 cycle; 95 °C 10 s, 58 °C 30 s, and 72 °C 34 s for 35 cycles followed by the melting curve stage. And the relative gene expression in each sample was recorded as the average 2^ − ΔΔCT calculation result of three replicates. Further, T-test was used for detailed statistical analysis. *P *< 0.05 was considered statistically significant.

### Association between the selected hub genes expression and ccRCC clinical pathological features

Ualcan has been a widely used integrated data-mining platform for analyzing cancer transcriptome data, besides previous analysis of the expression difference of hub genes in broad-spectrum human cancers comparing to corresponding normal control samples, UALCAN was in addition applied to analyze the association between hub genes’ expression and ccRCC clinical parameters including patients age, gender, tumor grade, stage and lymph node metastasis, aiming to aiding the better understanding of the potential biological roles of selected hub genes in ccRCC.

### Other genetic alterations of the selected hub genes as well as their potential related signaling pathways analysis

Besides the mRNA expression difference as well as association with clinical pathological parameters, the PPI networks which were centered on each of the four selected hub genes were constructed in succession, for the purpose of aiding better understanding of hub genes’ potential biological roles as well as their related signaling pathways by KEGG analysis in ccRCC development [40, 41].

Moreover, other types of variations of the selected key genes including mutation ratio, copy number variation, amplification and deletion ratio were explored based on cBioPortal database which has been an effective cancer genomics data website covering more than 2,8000 cancer samples. After logging into the cBioPortal website, the “cancer types summary” module of “quick search” section was used for exploring the genetic alteration characteristics of previous selected hub genes in various cancer types, especially ccRCC.

### Construction and clinical features analysis of an prognosis related immune gene signature

To maximum the clinical utilization of hub gene indicators that were selected based on above processes, an prognosis-related immune signature was constructed using LASSO algorithm performed with glmnet R package based on TCGA ccRCC data, thus an unique regression coefficient was assigned to each gene indicator which multiplies the gene expression. Based on the final score of each case calculated according to the gene signature, ccRCC patients were classified as high-risk and low-risk groups (median score was set as cut off value).

Further, the clinical features of high-risk and low-risk groups of patients were analyzed based on TCGA data which contains resourceful ccRCC samples, despite the censoring data, an effective pool of over 533 cases of ccRCC patients information were applied for preliminary observing the clinical features association of the constructed signature.

### Preliminary prognosis validation of the gene signature

To validate the survival relationship of last step constructed gene signature, a series of methods were combine used to analyze the TCGA ccRCC data including firstly Kaplan–Meier survival which was used to compare the survival difference between high-risk and low-risk groups of patients, then AUC curve was performed to observe the 1, 3 and 5 years survival prediction ability of the signature. Further, univariate as well as multivariate Cox regression analysis were applied for testing the independence survival prediction ability of the signature together with other well accepted prognosis related clinical parameters. Furthermore, a nomogram combining the signature and these validated clinical parameters was constructed for together evaluating clinical ccRCC patients prognosis, all based on the TCGA over 533 cases of ccRCC patients information.

Moreover, an independent GEO profile different from the four profiles that were used to identify the hub genes and construct the gene signature, namely GSE22541 was additionally applied to perform the Kaplan–Meier survival as well as ROC curve analysis, for the purpose of validating the prognosis correlation of the gene signature.

### Gene set enrichment analysis (GSEA) of two ccRCC patients subgroups based on gene signature

After preliminary prognosis relationship analysis, the gene signature was next step processed for further immune association evaluating. Based on the constructed gene signature, TCGA ccRCC patients were divided as high-risk and low-risk subgroups, for determining how the immunological pathways and corresponding immune genes differ between the two ccRCC subgroups, GSEA [42] was performed for signaling enrichment analysis, and the threshold was set as *P* < 0.05.

### Difference of immunogenic cell death (ICD) between high-risk and low-risk groups of ccRCC patients

ICD has been gradually accepted as a form of regulated biological cell death meanwhile supported by evidence-based medicine to be able to trigger cellular adaptive immune response through the emission of damage associated molecular patterns (DAMPs), thus potentially contributing to clinical immunotherapy. In the study, the expression distribution of 32 ICD related genes [43] which were identified based on literature studies were explored in high-risk and low-risk groups of ccRCC patients for preliminary evaluating the immune status difference between the two groups patients.

### Association between the gene signature and ccRCC estimated environment immune score

ESTIMATE, which is short for Estimation of Stromal and Immune cells in Malignant Tumors using Expression data and has been a well accepted cancer immune evaluation tool in multiple cancers was applied to estimate the immune score of ccRCC samples based on TCGA genes expression data. The correlation between the signature and ESTIMATE algorithm based ccRCC immune score, stromal score as well as tumor purity were evaluated using R package for aiding the validation of immune relationship of the constructed gene signature.

### Correlation between gene signature and the expression of immune checkpoint inhibitors

Immune checkpoints have been showing inspiring drug targeting effects in multiple cancers by reversing the tumor immuno suppressive microenvironment, and expression of immune checkpoints especially PD-L1, CTLA4, TIGIT, TIM-3 and LAG-3 have been well accepted as clinical biomarkers for selecting potential cancer patients that were most likely to benefit from immunotherapy. Therefore, the association between the gene signature and expression level of these immune checkpoints in TCGA ccRCC samples were assessed in the study, as well as the comparison of expression difference of these immune checkpoints between high-risk and low-risk groups of ccRCC patients.

### Evaluation of relationships between gene signature and 22 tumor infiltrating immune cells (TICs)

For characterizing the microenvironment immune landscape between high-risk and low-risk groups of ccRCC patients, CIBERSORT algorithm [44] was performed to calculate the relative contents of 22 TICs based on TCGA profiles data, followed by analyzing the relationship between the 22 TICs and gene signature. Further, the survival analysis of 22 TICs especially the ones that relates with gene signature were conducted for identifying the specific immune cell infiltration that impacts patients prognosis.

### Statistical analysis

Statistical analysis was performed using SPSS 26.0. For the enumeration data including QPCR experiment revealing the expression of different genes in ccRCC cancers vs normal control samples, the data were analyzed using t-test. As for the measurement data for instance the association between constructed gene signature and ccRCC clinical parameters, the data were analyzed by χ^2^ test. And for the correlation analysis, for instance the correlation between gene signature and immune checkpoints expression, the data were analyzed by Spearman analysis. Meanwhile, for the survival analysis, Kaplan–Meier were performed. *p* < 0.05 was considered statistically significant.

## Results

### ccRCC transcriptome data identified 269 high level differently expressed meanwhile immune related genes in cancer versus normal renal samples

**Fig. 1:**
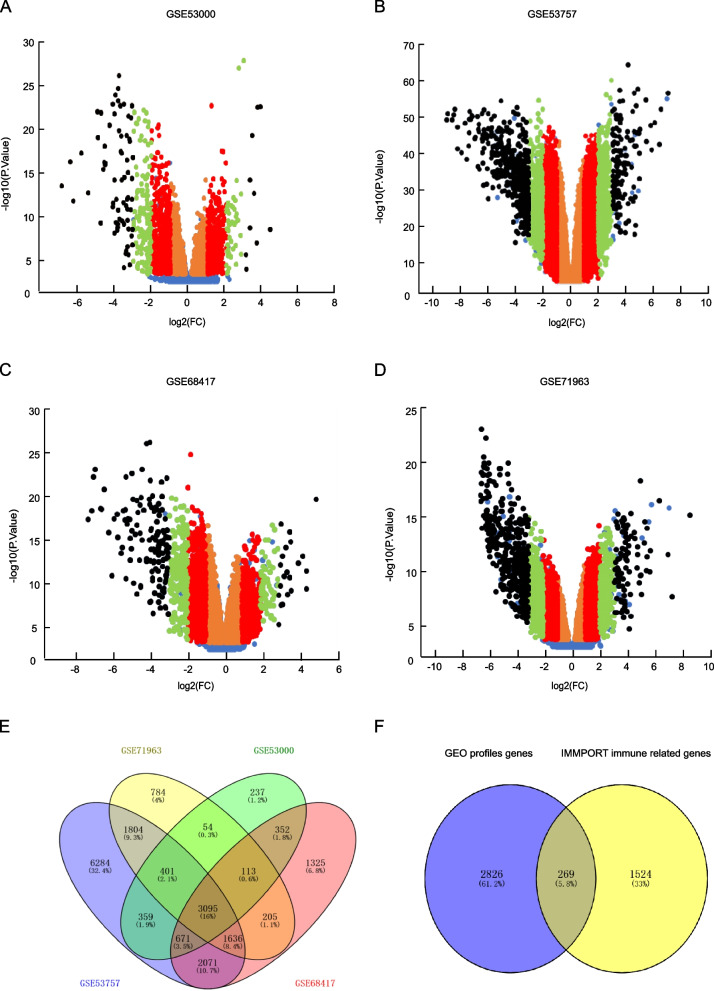
Differential expression genes in ccRCC comparing to normal renal samples identified from GEO profiles. Four GEO profiles (**A**) GSE53000, (**B**) GSE53757, (**C**) GSE68417 and (**D**) GSE71963 were accessed to identify differently expressed genes in ccRCC vs normal renal samples, and based on these profiles, the up-regulated (right side) and down-regulated (left side) differential expression genes in ccRCC were identified. The genes were then divided into four groups based on the expression difference level as: < twofold genes (orange-colored spots), 2 ~ fourfold genes (red-colored spots), 4 ~ eightfold genes (green-colored spots) and > eightfold genes (black-colored spots). **E** The intersection of the genes in four GEO profiles for revealing the genes that were shared in different profiles. **F** The intersection of differential expressed genes revealed by GEO profiles and immune related genes from IMMPORT database, thus the genes that were both differential expressed and immune related were revealed

Four GEO profiles GSE53000, GSE53757, GSE68417 and GSE71963 were combine applied to explore the aberrant differently expressed genes in ccRCC comparing to normal renal samples. And in GSE53000, a total of 5559 genes were identified to be differently expressed including 4270 genes with the expression change ≤ twofold, 1028 genes that were 2 ~ fourfold, 180 genes that were 4 ~ eightfold and 81 genes whose expression were > eightfold in ccRCC comparing to normal renal samples (Fig. 1A). And in GSE53757, a total of 28021 genes were identified, and the number was 21367, 4857, 1195 and 602 in ≤ twofold, 2 ~ fourfold, 4 ~ eightfold and > eightfold groups respectively (Fig. 1B). In GSE68417, a total of 10150 genes were identified, and the number was 8276, 1425, 290 and 159 in each group (Fig. 1C). Meanwhile, in GSE71963, a total of 10744 genes were identified, and the number was 6266, 3120, 841 and 517 genes in each group respectively (Fig. 1D, Table S2).

Considering the feasibility of further clinical medical use, we mainly focused on the high differently expressed genes (at least > fourfold in cancer vs. normal). As a result, besides the genes that were shared in multiple profiles, the analysis of 4 GEO profiles indicated a total of 3095 genes that were high level changed expressed in ccRCC comparing to normal control samples (Fig. 1E). Further, the immune related genes list was obtained from IMMPORT immune database, and Venn diagram analysis result identified 269 genes from the 3095 genes that were both high level expression changed and immune regulation related for next step analysis (Fig. 1F, detailed 269 genes information was listed in Table S3).

### PPI network of 269 genes highlighted a 46 genes-containing immune relating gene cluster

**Fig. 2:**
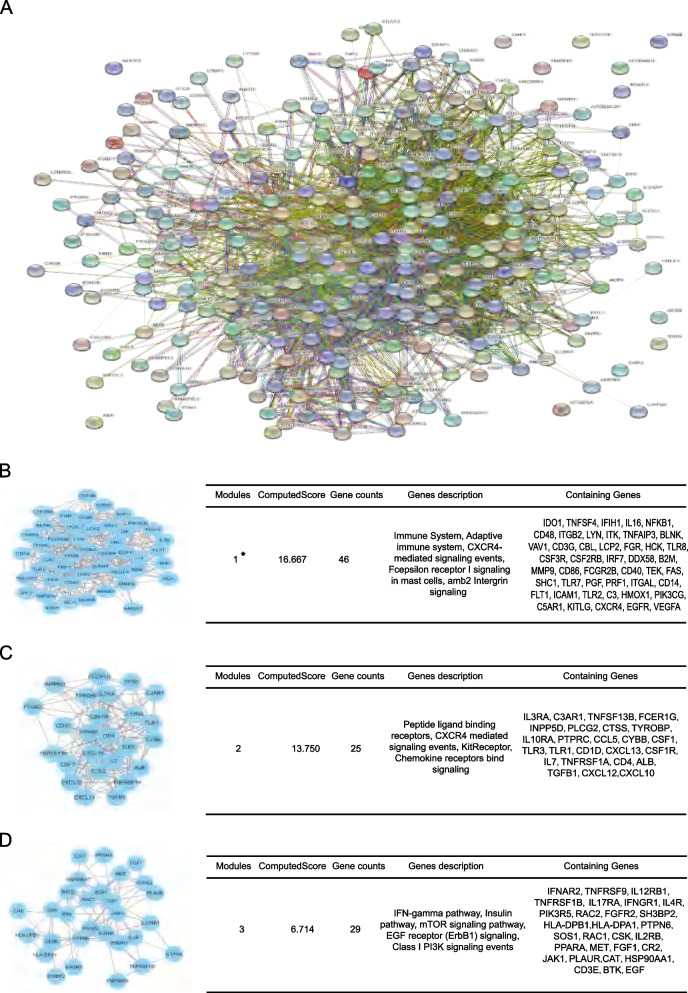
PPI network construction of 269 differential expressed meanwhile immune related genes in ccRCC and function modules analysis. (**A**) Based on four GEO profiles as well as IMMPORT datasets, 269 differential expression meanwhile immune related genes in ccRCC were identified, and the PPI network of these 269 genes was constructed. And based on the PPI network, (**B**) the first, (**C**) second and (**C**) third genes function modules were analyzed, each module was shown with a diagrammatic sketch (left diagram) and the detailed module information (right table) including the computed module score, module description and detailed involving genes. (*The first module with the highest module score meanwhile immune regulation related was focused for further analysis)

The PPI network of 269 differently expressed meanwhile potentially immune related genes was constructed (Fig. 2A), and based on the network we identified three promising gene clusters. The first gene cluster posses the highest computed module score and contains 46 genes with a big portion of them predicted to be related with immune system modulation (Fig. 2B). Meanwhile, the second and third gene modules contain 25 and 29 genes respectively, and genes were mostly related with CXCR4, PI3K, EGF and mTOR related signaling pathways (Fig. 2C, D).

Given the first module gene cluster possess the highest score and a big percentage of containing genes were immune system related which shows more potential for further clinical immune indicators selection, the 46 genes in the gene cluster were mainly focused for next step analysis.

### Kaplan–Meier combine with Cox regression analysis of cluster genes identified 4 ccRCC prognosis related hub genes

**Fig. 3:**
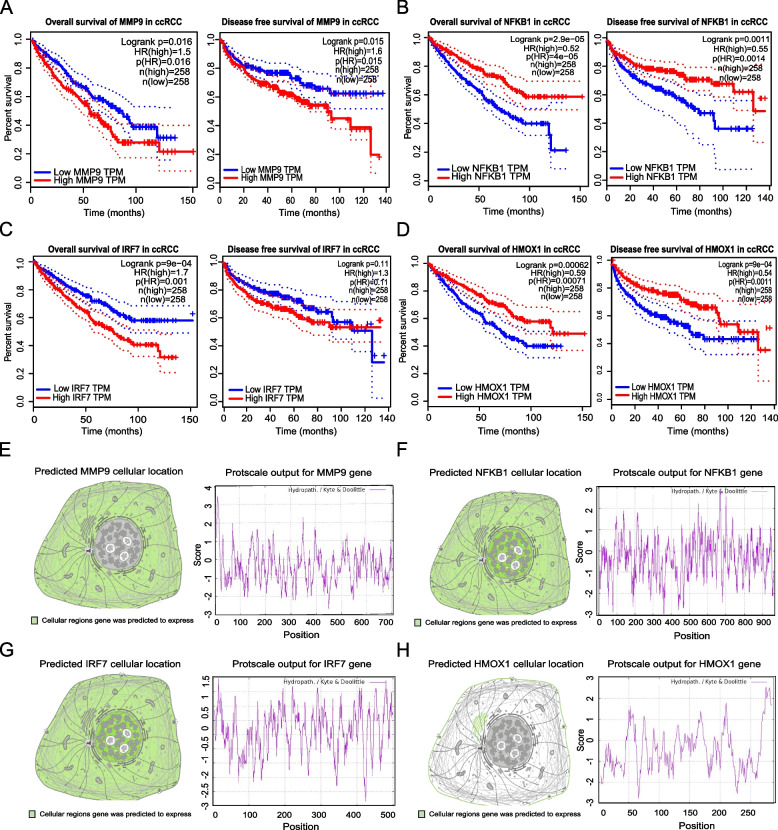
Survival analysis and basic physicochemical properties exploration of four selected signature comprised genes. The overall survival (left) and disease free survival (right) analysis in ccRCC patients, including (**A**) MMP9 gene, (**B**) NFKB1 gene, (**C**) IRF7 gene and (**D**) HMOX1 gene. The predicted cellular location (left) and computed hydrophility/hydrophobicity property of (**E**) MMP9 protein, (**F**) NFKB1 protein, (**G**) IRF7 protein and (**H**) HMOX1 protein

Genes should be of more potential if they were both immune regulation and survival related, and promising immune biomarkers should also be prognosis related for potential clinical medical drug targeting use. To further analyze survival relationship of the 46 selected immune related candidate genes, univariate survival analysis including UALCAN and GEPIA, as well as multivariate Cox regression analysis were in succession performed, and the results supported four genes, namely MMP9 (Fig. 3A), NFKB1 (Fig. 3B), IRF7 (Fig. 3C) and HMOX1 (Fig. 3D) as independent prognostic indicators in ccRCC, all four genes not only relate with patients overall survival but also progress free survival indicating their high potential in clinical medical use (Table 1).

**Table 1:** Univariate combine with multivariate Cox Regression analysis result of the 4 hub genes used for signature construction

OSCC parameters	*P* Value			B value	HR	95% CI
	**Univariate analysis**		**Multivariate analysis**			
	UALCAN	GEPIA				
MMP9	0.001	0.016	0.002	0.256	1.292	1.009–1.519
NFKB1	0.013	2.9E-05	0.004	-0.216	0.805	0.696–0.932
IRF7	< 0.0001	9E-04	0.022	0.077	1.080	1.011–1.154
HMOX1	0.034	0.00062	< 0.001	-0.222	0.801	0.708–0.906

### Basic physicochemical properties of the 4 selected hub genes

Basic physiochemical properties of MMP9, NFKB1, IRF7 and HMOX1 were preliminary interpreted before deeper scientific use of them (Table 2). As for MMP9, which is a member of matrix metalloproteinase (MMP) family, locates in 20q13.12 and encodes a protein composed of 707 amino acids with an estimated molecular weight of 78.5KD. The theoretical isoelectric point of the protein is estimated to be 5.69 and instability index to be 41.10, meanwhile, the grand average of hydrophobic value of the protein is -0.394 indicating MMP9 works as a cellular unstable and hydrophilic protein which locates in cellular cytoplasm or to be secreted in the extracellular region, and the related signaling pathways include collagen chain trimerization and apoptotic pathways in synovial fibroblasts (Fig. 3E).

**Table 2:** Basic physicochemical properties of the 4 hub genes used for gene signature construction

Gene Property	MMP9	NFKB1	IRF7	HMOX1
Formula	C_3517_H_5298_N_958_O_1035_S_28_	C_4643_H_7343_N_1271_O_1458_S_33_	C_2418_H_3740_N_678_O_710_S_19_	C_1475_H_2323_N_405_O_427_S_8_
Molecular Weight	78.46KD	105.36KD	54.28KD	32.82KD
Number of amino acids	707AA	968AA	503AA	288AA
Theoretical pI	5.69	5.20	5.89	7.89
Aliphatic index	65.13	84.74	72.07	83.02
Hydrophobic value	-0.394	-0.339	-0.367	-0.427
Estimated protein half life	30 h	30 h	30 h	30 h
Instability index	42.10	38.15	63.17	60.81

NFKB1, which is short for Nuclear Factor Kappa B Subunit 1 locates in 4q24 and encodes a protein composed of 968 amino acids and estimated to be weighting 105KD with computed theoretical isoelectric point as 5.20 and instability index as 38.15. Meanwhile, the grand average of hydrophobic value of protein is -0.339 indicating NFKB1 to be cellular stable and hydrophilic. NFKB1 is predicted to locates in nucleoplasm and cytoplasm, it has been reported as a transcription regulator that could be activated by various cellular stimuli such as cytokines, ultraviolet irradiation, and bacterial or viral products. Activated NFKB1 translocates into cell nucleus and stimulates the expression of genes involved in various biological functions (Fig. 3F).

IRF7 is short for Interferon Regulatory Factor 7 and it’s a member of the interferon regulatory factor (IRF) family, locating in 11p15.5 and encoding a protein composed of 503 amino acids including 56 negatively charged amino acid residues (ASP + Glu) and 49 positively charged amino acid residues (Arg + Lys). The estimated protein molecular weight is 54.2KD with theoretical isoelectric point computed to be 5.89. Meanwhile, the estimated instability index of the protein is 63.17 and grand average of hydrophobic value is -0.367, the cellular location of the gene is predicted to be in nucleoplasm or cytoplasm (Fig. 3G).

Meanwhile, HMOX1 is short for Heme Oxygenase1, and locates in 22q12.3, encoding a protein composed of 288 amino acids including 35 negatively charged amino acid residues (ASP + Glu) and 36 positively charged amino acid residues (Arg + Lys). The estimated protein molecular weight is 32.8KD with theoretical isoelectric point computed as 7.89. Moreover, the estimated instability index of the protein is 60.81 and grand average of hydrophobic value is -0.427 indicating HMOX1 works as a cellular unstable and hydrophilic protein which is consistent with the ProtScale analysis result of HMOX1 structure showing that the protein harbors more hydrophilic regions than hydrophobicity regions. HMOX1 is predicted to locates in cellular Golgi apparatus and plasma membrane, it has been reported to be associated with the development of heme oxygenase1 deficiency and pulmonary disease, as well as chronic obstructive (Fig. 3H).

### Validation of the changed expression of selected hub genes in ccRCC versus normal renal tissues

Although the four selected hub genes were obtained from the differently expressed gene clusters analyzed based on GEO data from the beginning, after preliminary interpretation of the basic physicochemical information, it’s necessary to validate each of the gene’s aberrant changed expression in ccRCC comparing to normal renal samples individually. In the study, both online analysis as well local hospital samples were used for detecting the genes expression level.

**Fig. 4:**
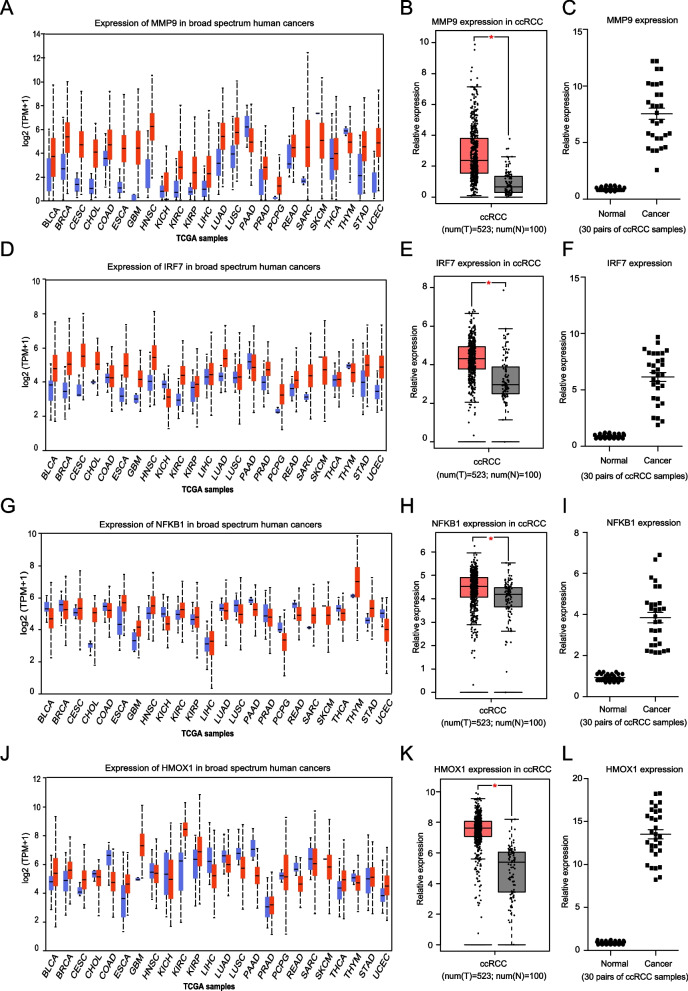
The differential expression of four selected signature comprised genes in ccRCC included human cancers. UALCAN prediction of (**A**) MMP9 gene, (**D**) IRF7 gene, (**G**) NFKB1 gene and (**J**) HMOX1 gene expression in broad spectrum human cancers. GEPIA analysis of (**B**) MMP9 gene, (**E**) IRF7 gene, (**H**) NFKB1 gene and (**K**) HMOX1 gene in ccRCC comparing to normal renal samples. QPCR experiment using local hospital ccRCC samples for validating the changed expression of (**C**) MMP9 gene, (**F**) IRF7 gene, (**I**) NFKB1 gene and (**L**) HMOX1 gene in ccRCC comparing to normal renal samples

Firstly, two analysis databases including UALCAN and GEPIA were used, and the results revealed that as for MMP9 and IRF7 genes, they gain of expression in most of human cancers (Fig. 4A, D). And as for NFKB1 and HMOX1, their expression vary in different cancers (Fig. 4G, J), although in ccRCC, all four genes were indicated to be statistical significantly up regulated in cancers comparing to normal renal tissues (Fig. 4B, E, H, K).

Then, the result of qRT-PCR experiment which was conducted using 30 local hospital ccRCC and paired normal renal tissues also supported the aberrant gain of expression of all four genes including MMP9, IRF7, NFKB1 and HMOX1 in ccRCC (Fig. 4C, F, I, L).

### Correlation analysis between the selected hub genes and ccRCC clinical parameters

**Fig. 5:**
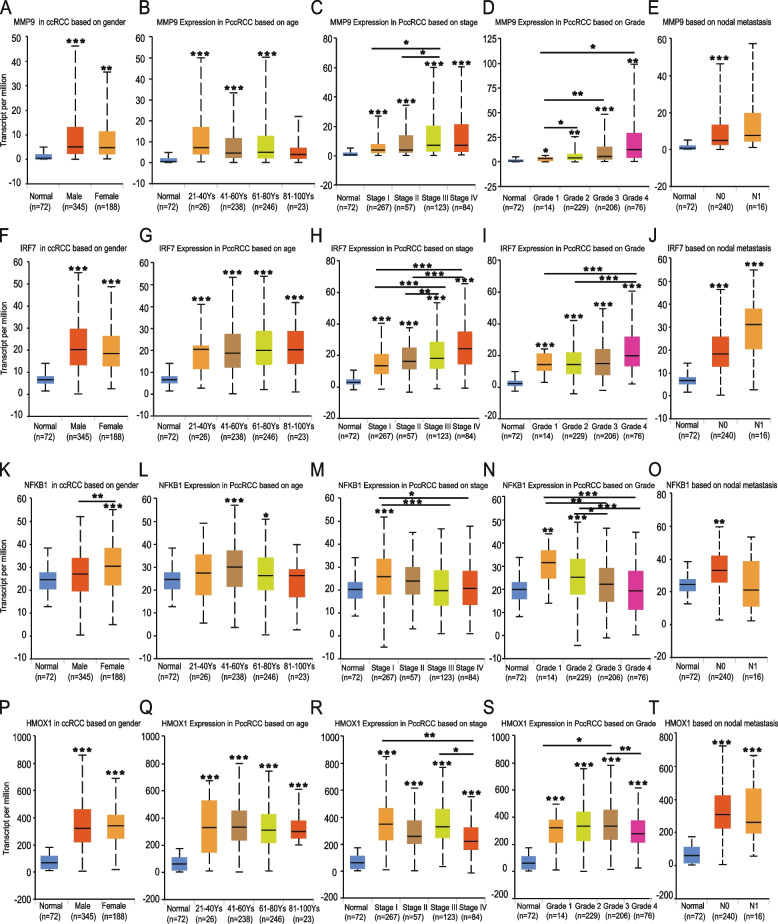
The association between four selected hub genes expression and ccRCC clinical parameters. The association between MMP9 expression and ccRCC (**A**) patients gender, (**B**) age, (**C**) cancer stage, (**D**) cancer grade and (**E**) lymph node metastasis. The association between IRF7 expression and ccRCC (**F**) patients gender, (**G**) age, (**H**) cancer stage, (**I**) cancer grade and (**J**) lymph node metastasis. The association between NFKB1 expression and ccRCC (**K**) patients gender, (**L**) age, (**M**) cancer stage, (**N**) cancer grade and (**O**) lymph node metastasis. The association between HMOX1 expression and ccRCC (P) patients gender, (**Q**) age, (**R**) cancer stage, (**S**) cancer grade and (**T**) lymph node metastasis. (* *p* < 0.05, **p < 0.01, ***p < 0.001. The first layer * which is right above the error bar representing comparison to normal group, and the above layers * which were above a secondary line represent the comparison between corresponding groups that were covered by the line)

For analyzing the association between MMP9, IRF7, NFKB1 and HMOX1 expression and ccRCC clinical parameters, UALCAN online platform was used. And the result revealed an inspiring fact that although all four genes were indicated to express higher in cancer comparing to normal samples, the four genes tend to play opposite roles in cancer development. To be more specific, the expression of MMP9 and IRF7 genes which were supported by previous survival analysis to be related with worse patients prognosis tend to be higher as the cancer stage and grade advancing, more obviously, both of the genes express higher in patients with node metastasis (Fig. 5A-J).

Meanwhile, as for NFKB1 and HMOX1 genes which were indicated to relate with better patients prognosis, the expression tend to keep decreasing as the cancer stage and grade advancing, and their expression were lower in patients with lymph node metastasis, although the difference was not statistical significant presumably due to the limited patients cases number in N1 group (Fig. 5K-T).

### Selected hub genes mainly enriched signaling pathways and other types of genetic alteration analysis

**Fig. 6:**
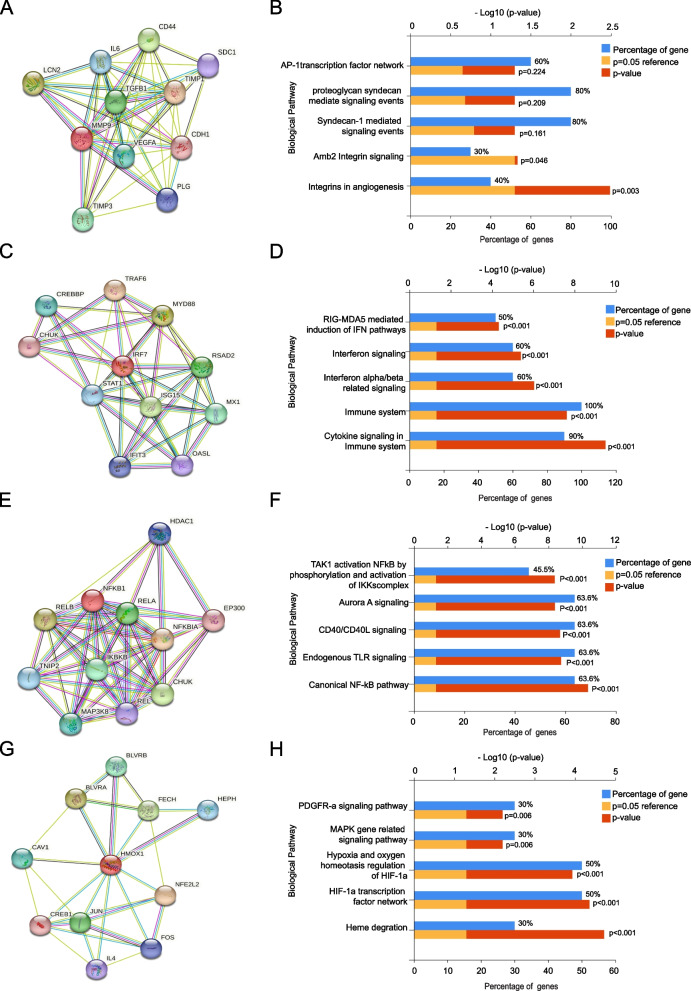
PPI network centered on four selected hub genes and GO/KEGG analysis of their enriched biological pathways. (**A**) The PPI network which is centered on MMP9 gene for analyzing (**B**) the main biological signaling pathways MMP9 and its connected genes mainly participated in. (**C**) The PPI network which is centered on IRF7 gene for analyzing (D) the main biological signaling pathwaysIRF7 and its connected genes mainly participated in. (**E**) The PPI network which is centered on NFKB1 gene for analyzing (**F**) the main biological signaling pathways NFKB1 and its connected genes mainly participated in. (**G**) The PPI network which is centered on MMP9 gene for analyzing (**H**) the main biological signaling pathways MMP9 and its connected genes mainly participated in. (KEGG software analysis permitted by Kanehisa laboratory)

For preliminary exploring the mechanism behind the different roles of the four genes in ccRCC, the PPI network that were centered on each of the four genes including MMP9 (Fig. 6A), IRF7 (Fig. 6C), NFKB1 (Fig. 6E) and HMOX1 (Fig. 6G) were constructed, followed by GO/KEGG analyzing the biological functions as well as signaling pathways the fours genes and their surrounding partner genes most enriched.

And the results revealed that the four genes potentially evolved in different signaling in cancer development, as for MMP9, it mainly evolves in proteoglycan syndecan as well as integrin mediated signaling pathways (Fig. 6B), and IRF gene was indicated to be relate with immune and interferon related biological functions (Fig. 6D). Meanwhile, NFKB1 gene was related with the canonical NF-kB signaling as well as Aurora A, CD40/40L and endogenous TLR signaling pathways (Fig. 6F). And HMOX1 was indicated to be associated with HIF-1a gene related cancer hypoxia and oxygen homeostasis regulation (Fig. 6H).

Besides mRNA expression and potential related signaling pathways, other genetic alterations including mutation ratio, protein structure variant and copy number variation of the four genes were preliminary explored based on cBioPortal database. However, limited inspiration could be achieved based on this part of analysis considering the fact that only few ccRCC samples were included in cBioPortal database, although various types of MMP9 (Figure S1A), IRF7 (Figure S1B), NFKB1 (Figure S1C) and HMOX1 (Figure S1D) genes alteration were observed in different human cancers indicating the potential different functions these genes play in human cancers.

### Construction of a 4 genes containing ccRCC prognosis related immune gene signature and clinical features analysis

**Fig. 7:**
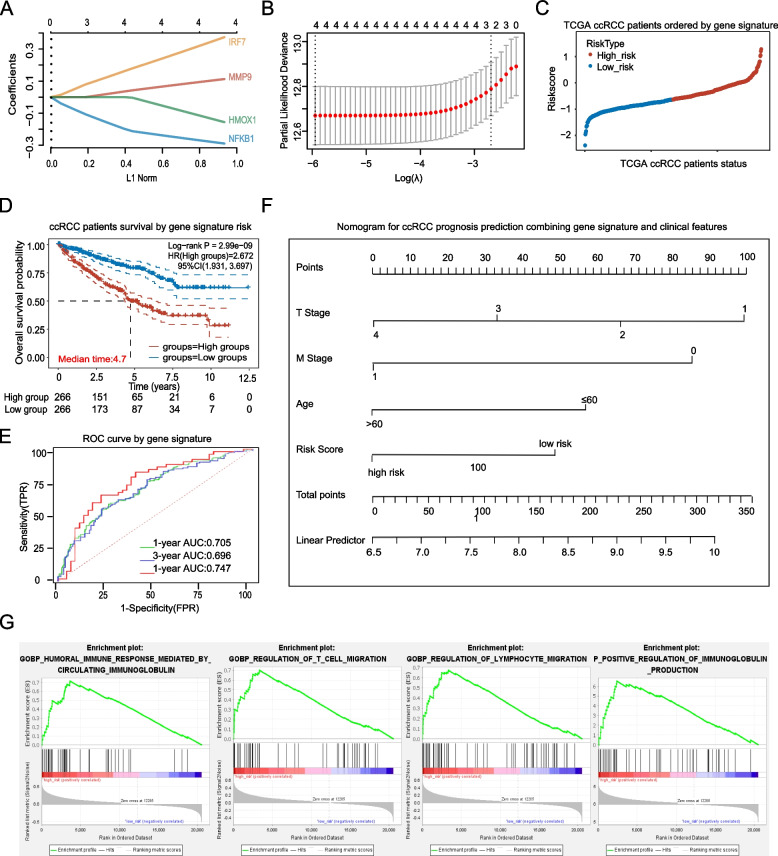
Construction of a four genes containing meanwhile immune and prognosis related ccRCC gene signature**.** LASSO analysis to calculate (**A**) the coefficient and (**B**) the likelihood deviance for constructing a suitable immune meanwhile prognosis related signature which was comprised of strictly calculated four genes. (**C**) TCGA ccRCC patients were divided into high-risk and low-risk groups based on the calculated signature score (the cut off value was set as the median signature score in all samples). (**D**) Survival analysis of the high-risk and low-risk groups of ccRCC patients. (**E**) ROC curve of the gene signature to predict ccRCC patients survival of 1 year, 3 years and 5 years respectively. (**F**) ccRCC patients prognosis prediction nomogram constructed based on genes signature and clinical parameters which were supported by Cox Regression to be independently related with patients survival. (**G**) Significant enrichment of immune-related phenotype including immune response and immune cells migration in high-risk group of ccRCC patients compared with that in low-risk group patients

To maximum the clinical prediction value of the four selected genes, Cox-proportional hazards analysis based on LASSO algorithm was applied to construct a MMP9, NFKB1, IRF7 and HMOX1 four genes containing signature which weights the normalized expression level of each gene to the regression coefficient of multivariate Cox regression analysis. And the result revealed a formula: Risk Score = 0.256 * expression (MMP9)—0.222 * expression (HMOX1)—0.216 * expression (NFKB1) + 0.077 * expression (IRF7) as the best signature for combining the four differently expressed meanwhile immune related hub genes (Fig. 7A, B).

Based on the signature, the risk score for each patient was calculated followed by the patients being categorized into high-risk or low-risk groups according to the median risk score which was set as the cut off point for the signature (Fig. 7C). Further, the association between the gene signature and ccRCC clinical features was preliminary analyzed, which result revealed that higher risk score was positively related with older age (> 45 years old) and more advanced T, N and M stage, meanwhile, the low risk group of patients were tend to be younger (≤ 45 years old) female with lower TNM stage (Table 3).

**Table 3:** Association between the gene signature and ccRCC clinical features

Parameters	Gene signature		*P* Value
	Low-risk group	High-risk group	
Gender			
female	112 (59.6%)	76 (40.4%)	0.001
male	154 (44.6%)	191 (55.4%)	
Age			
≤ 45	38 (64.4%)	21 (35.6%)	0.018
> 45	228 (48.1%)	246 (51.9%)	
Race			
White	234 (50.6%)	228 (49.4%)	0.697
Yellow	4 (50.0%)	4 (50.0%)	
Black	25 (44.6%)	31 (55.4%)	
T stage			
T1	164 (60.01%)	109 (39.9%)	< 0.001
T2	31 (44.9%)	38 (55.1%)	
T3	69 (38.3%)	111 (61.7%)	
T4	2 (18.2%)	9 (81.8%)	
N stage			
N0	115 (47.9%)	125 (52.1%)	0.023
N1	3 (18.8%)	13 (81.3%)	
M stage			
M0	231 (54.7%)	191 (45.3%)	< 0.001
M1	22 (27.8%)	57 (72.2%)	

### High risk score based on the gene signature indicated worse ccRCC patients prognosis

All four genes used to construct the gene signature were previously supported to be high level differently expressed in ccRCC comparing to normal renal samples, but changed gene expression doesn’t equal to survival association. To validate the survival relationship of the gene signature, two independent analysis methods were performed.

Firstly, a series of analysis were applied using TCGA ccRCC data including at first Kaplan–Meier survival analysis, which result revealed that the high risk group of patients had a statistical significantly worse overall survival than their low risk counterparts (Fig. 7D). Then the ROC curve showed that the area under the ROC curve (AUC) of gene signature for overall survival was 0.747 at 1 year, 0.696 at 3 year and 0.705 at 5 years (Fig. 7E). Meanwhile, univariate Kapkan-Meier survival as well as multivariate Cox regression analysis were applied for testing the survival prediction ability of the signature, and the results supported the risk score calculated based on the gene signature works as an independent prognosis indicator for ccRCC patients together with some other well accepted prognosis related clinical parameters including patient T and M stage (Table 4). Further, a nomogram was constructed and and in the nomogram, a point scale was assigned for each variable, the sum of all the variables points equal to the final score of each patient, and the survival could be predicted by drawing a vertical line from the total point axis downward to the outcome axis (Fig. 7F).

**Table 4:** Survival prediction value of the gene signature included ccRCC clinical parameters

Clinical parameters	*P* Value		Exp (B)
	**Univariate analysis**	**Multivariate analysis**	
Age	< 0.001	0.026	1.627 (1.058–2.500)
Gender	0.693	-	-
Race	0.719	-	-
T stage	< 0.001	0.018	1.360 (1.054–1.755)
N stage	< 0.001	0.480	1.290 (0.636–2.615)
M stage	< 0.001	< 0.001	2.794 (1.728–4.517)
Signature risk score	< 0.001	0.002	1.871 (1.299–2.693)

Secondly, besides above TCGA data, an independent GEO ccRCC cDNA expression profile GSE22541 was also included for validating the prognosis correlation of the gene signature. After observing the detailed expression of all the four genes in the GSE22541 datasets (Figure S2E), the patients samples were divided as high and low risk groups based on the constructed gene signature score (Figure S2A, S2B). Afterwards, Kaplan–Meier survival (Figure S2C) as well as ROC curve (Figure S2D) were also conducted, and the results supported the high risk group of patients had a statistical significantly worse prognosis than low risk group patients.

### High-risk group of ccRCC cases were more enriched in immune related phenotype

After preliminary demonstrating the association between constructed gene signature and ccRCC prognosis, the influence of gene signature on cancer immune profiles was to be investigated. And in the first step, GSEA was utilized to analyze the immune-related biological processes linked to the signature, and the analysis result showed that the high-risk group cases were significantly enriched in multiple biological processes, of which 4 immune-related processes were identified including HUMORAL_IMMUNE_RESPONSE (NES = 1,733, Nominal *p* value = 0.0), REGULATION_OF_T_CELL_MIGRATION (NES = 1.762, Nominal *p* value = 0.0), REGULATION_OF_LYMPHOCYTE_CELL_MIGRATION (NES = 1.743, Nominal *p* value = 0.003), POSITIVE_REGULATION_OF_IMMUNOGLOBULIN_PRODUCTION (NES = 1.754,Nominal *p* value = 0.0). Meanwhile, the low-risk group cases were not indicated to be enriched in any immune-related biological processes (Fig. 7G).

### High-risk and low-risk groups of ccRCC patients revealed disparate ICD expression levels

**Fig. 8:**
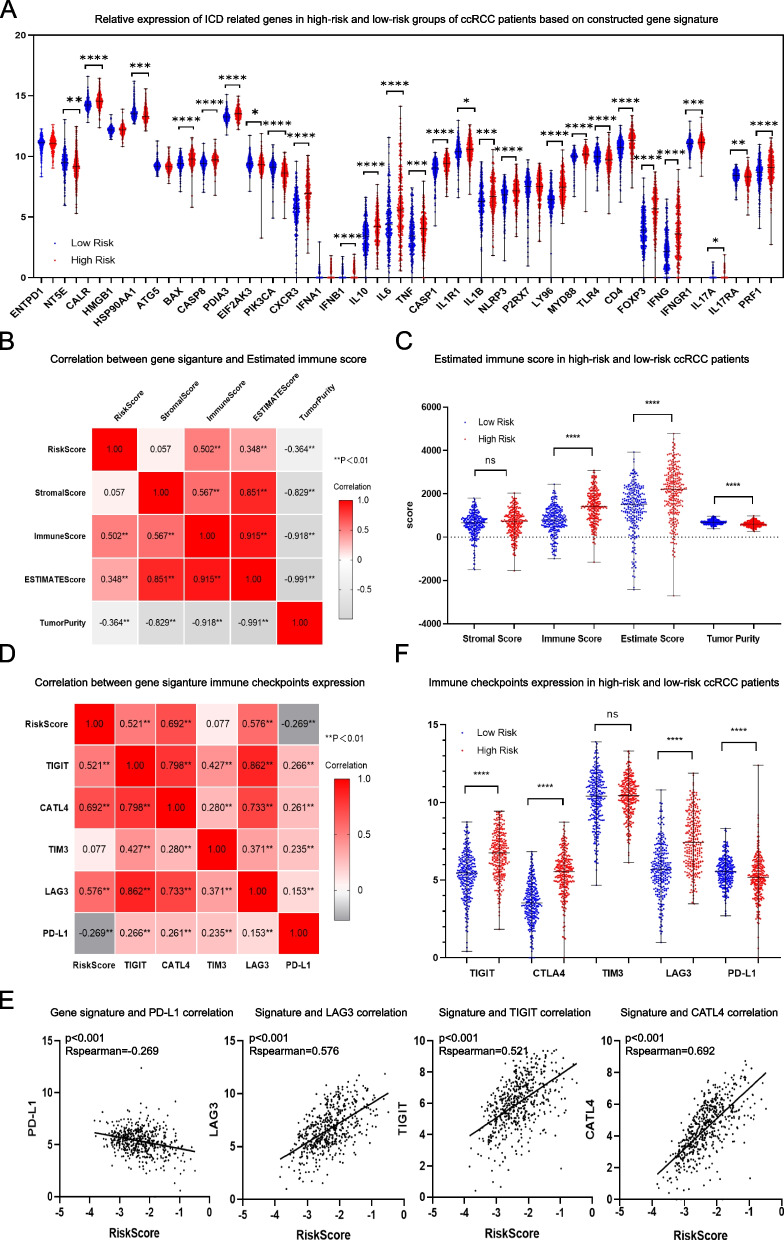
Correlation between gene signature and ccRCC immune microenvironment landscape. (**A**) Relative expression of ICD related genes in high-risk and low-risk groups of ccRCC patients. (**B**) Correlation between gene signature and ccRCC computed immune score, stromal score and tumor purity calculated using ESTIMATE algorithm. (**C**) Estimated immune score, stromal score and tumor purity distribution in high-risk and low-risk ccRCC groups respectively. (**D**) Association between gene signature and immune checkpoints expression. (**E**) Correlation between gene signature and PD-L1, LAG-3, TIGIT and CALT-4 expression respectively. (**F**) Relative expression of five immune checkpoints including PD-L1, LAG-3, TIGIT, TIM3 and CALT-4 expression in high-risk and low-risk ccRCC groups respectively

Besides GSEA immune phenotype enrichment analysis, given the significant roles of ICD in antitumor immunological responses, the connection between gene signature and ICD related genes were evaluated for additionally exploring the immune status in high-risk and low-risk groups of ccRCC patients. And the results revealed that the expression of a large portion of the 32 ICD related genes were statistical significantly different between the two groups of patients indicating the diverse immune status in the microenvironment of two groups of patients (Fig. 8A).

### Risk score calculated based on the gene signature associated with ccRCC estimated environment immune score

For further validating the immune association of the gene signature, ESTIMATE was performed to evaluate the immune, stromal score and tumor purity of ccRCC samples. And the result revealed that although no significant correlation was found between the gene signature and ccRCC stromal score, a mediate correlation was revealed between the risk score which was calculated based on the gene signature and tumor immune score as well as tumor purity (Fig. 8B). Meanwhile, the high risk-group patients were tend to posses higher immune score and lower tumor purity, indicating the immune targeting potential of the group of patients (Fig. 8C).

### Multi immune checkpoints expressed higher in high risk group of ccRCC patients based on the gene signature

Besides estimation of immune score, the association between the gene signature and clinical promising immune checkpoints including PD-L1, CTLA4, TIGIT, TIM-3 and LAG-3 were evaluated (Fig. 8D). And a median association was revealed between the gene signature and CTLA4 expression. Moreover, mild correlation was indicated between gene signature and two of the immune checkpoints including LAG3 and TIGIT, meanwhile, no significant relation was found between the signature and PD-L1 or TIM-3 expression (Fig. 8E). An inspiring fact was that all CTLA4, LAG3 and TIGIT tend to express higher in high-risk group of patients which was categorized based on the gene signature (Fig. 8F), and the distribution shall be an additional support besides above ESMINATE immune score evaluation result for indicating the immune targeting potential for this group of ccRCC patients.

### Evaluation of relationships between gene signature and 22 tumor infiltrating immune cells (TICs)

**Fig. 9:**
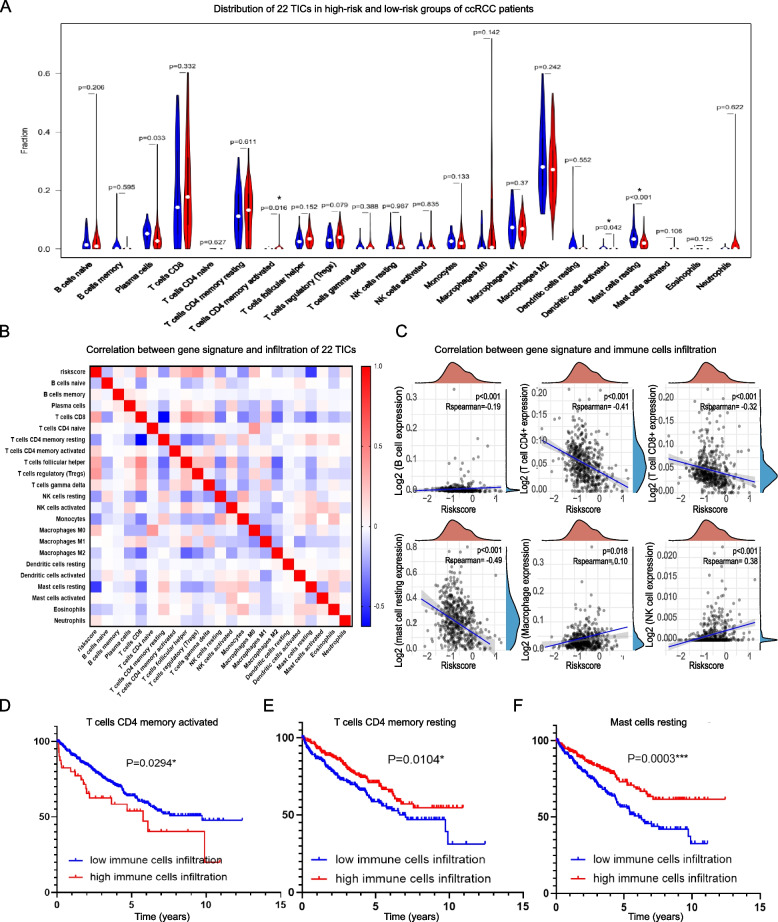
Correlation between gene signature and 22 immune cells infiltration in ccRCC. (**A**) Relative distribution of 22 immune cells in high-risk and low-risk groups of ccRCC patients. (**B**, **C**) Correlation between gene signature and various immune cells infiltration in ccRCC. Association between (**D**) T cells CD4 memory activated, (**E**) T cells CD4 memory resting and (**F**) Mast cells resting microenvironment infiltration and ccRCC patients survival

Previous analysis supported that the constructed gene signature was related to immunity, so we carried out analyses on 22 TICs whose distribution profiles were draw based on CIBERSORT algorithm to further study the interaction between the gene signature and ccRCC immune microenvironment. And the correlation analysis result found four types of TICs to be related with the gene signature including plasma cells, activated CD4( +) T memory cells, activated dendritic cells and resting mast cells (Fig. 9A-C).

Further, the prognostic abilities of the 22 TICs were tested and the results revealed that of the four signature related TICs, CD4( +) T cell and resting mast cell were able to predict ccRCC patients prognosis. The resting and activated CD4( +) T memory cells played opposite roles in patients survival, namely the activated CD4( +) T memory cells were related with worse patients survival (Fig. 9D), meanwhile, the resting CD4( +) T memory cells predicting better patients survival (Fig. 9E). Also, the resting mast cells were correlated with positive patients prognosis (Fig. 9F).

Combining the analysis results, one inspiring deduction could be draw that CD4( +) T memory cells and resting mast cells not only are significantly related to the gene signature but also predict ccRCC patients prognosis, indicating these immune cells may play important roles in the immune regulation of the gene signature in ccRCC microenvironment.

## Discussion

ICIs has been an increasing rising up clinical method and holds great promise for treating ccRCC [9], but the effective biomarkers for predicting immune response are still lacking, the well accepted immune prediction biomarkers in other cancers for instance PD-L1 expression, MSI status and TMB haven’t been supported thoroughly by evidence-based medicine to be effective in ccRCC [45, 46]. For the clinical benefit from ICI therapy, it is of great importance to keep exploring ccRCC genome and identifying new potential biomarkers thus benefiting further clinical application of immunotherapy in the cancer.

In recent years, multiple genes containing signatures representative of caner immune status have been identified in several cancers besides ccRCC, including gene signatures that were comprised of LncRNAs, miRNAs or immune regulation related genes, and they have been showing inspiring clinical effects [47–50]. Based on these reports, it’s of clinical feasibility to explore ccRCC genome information and develop meaningful immune prediction models which were also prognosis related to evaluate the immune status of ccRCC microenvironment and stratify patients into different groups for increasing the potential effectiveness of ICIs therapy. Considering most biological cellular functions were performed by different types of cell proteins, in the study, we mainly focused on the protein encoding genes that were aberrant differently expressed meanwhile prognosis as well as immune related for series of analysis. Thus, we mainly focused on GEO transcriptome profiles for identifing potential immune regulation related gene candidates.

Based on four different ccRCC cDNA expression profiles which were all selected by strict criteria as see in the Materials and Methods part, we identified the differential expression genes in ccRCC cancer vs. normal renal tissues and then divided them into 4 groups according to the difference level as < twofold, 2 ~ fourfold, 4 ~ eightfold and > eightfold genes considering the potential unique functions and clinical use of each group, for example, an interesting phenomenon has been discovered that the more genes expression difference are, the more their cellular location tend to be far away from cell nuclear [51–53]. For the feasibility of further clinical medical use, in the selection process of candidate genes, we mainly focused on the high level differently expressed namely at least > fourfold genes that were more convenient to be tested by IHC experiment which has been a common method in clinical pathology diagnosis, considering that the genes shall harbor more chance to be translated into clinical use if they are suitable to be tested by IHC. Further, the intersection between GEO selected high level aberrant differently expressed genes and immune related gene list from IMMPORT database indicated 269 genes that were both high level expression changed in ccRCC and immune related as candidate genes for next step analysis.

As for the construction of multi genes containing signature, LASSO algorithm has been widely accepted as an effective tool that is suitable to construct gene models basing on large numbers of correlated covariate. But instead of constructing an gene signature directly from the 269 candidate genes, we further in succession performed module analysis as well as multiple survival analysis to scale down the candidate genes and identify the promising “unique key genes” during ccRCC development, and only used LASSO for estimating the coefficient of signature genes. This is for the considering that the signature and consist genes should be of more clinical potential if they were not only immune regulation but also survival related for clinical medical drug targeting use. Therefore, the PPI network of the 269 genes was constructed followed by genes module analysis which highlighted a 46 genes containing cluster, and further survival analysis including GEPIA and UALCAN univariate survival as well as multivariate Cox Regression analysis of each of the 46 genes supported four genes: MMP9, NFKB1, IRF7 and HMOX1 to be associated with patients survival and worked as independent prognostic indicators in ccRCC development.

Interestingly, no direct relationship has yet been discovered among the four genes. MMP9 is s a member of matrix metalloproteinase (MMP) family which is well known to be involved in the breakdown of extracellular matrix during multiple normal physiological and diseases processes, and MMP9 has been reported to be able to degrades type IV and V collagen which are important microenvironment elements. NFKB1 is a transcription regulator that could be activated by cellular stimuli such as cytokines, ultraviolet irradiation, bacterial and viral products, and inappropriate activation of the gene has been known to associate with a number of inflammatory diseases, while persistent inhibition of NFKB1 leads to inappropriate immune cell development or delayed cell growth. IFR7 is predicted to locates in nucleoplasm and cytoplasm and it has been reported to play roles in innate immune response against DNA and RNA viruses. Meanwhile, HMOX1 is predicted to locates in cellular Golgi apparatus and plasma membrane and it has been reported to be associated with the development of heme oxygenase1 deficiency and pulmonary disease, as well as chronic obstructive. Moreover, the PPI network centered on the four genes and following KEGG analysis also supported the independent roles of these genes in cancer development. The together identification of the four genes and a gene signature combine all of them indicating the elaborate collaboration network of various genes in cellular activities, opening up further cancer researches of unlimited possibilities.

Based on the selected four genes and coefficient calculated with LASSO algorithm for each gene, an immune meanwhile prognosis related gene signature was constructed. Survival relationship validation including Kaplan–Meier survival, Cox proportional-hazards model and ROC curve based on both TCGA data and an independent GEO profile all supported the signature worked as an prognostic factor after combining the four genes in one equation, proving the effectiveness of apply the gene signature in ccRCC prognosis prediction. Since many clinical parameters especially tumor TNM stage as been well known as critical survival related aspects, we proposed a nomogram assessment that combines the signature and other clinical features. Although current result has not supported the signature to be a better prognosis factor than TNM stage, the construction of the nomogram shall work as a complementary perspective on individual tumour and aiding the comprehensive evaluation of clinical ccRCC patients prognosis.

Although survival relation was an important part, the main aim of the signature was for potential immune prediction. Immune escape has been one of the major characteristics in malignant tumors involving multiple probable mechanisms [54, 55], for example the increasing immune suppressive cells including Treg cells and tumour-associated macrophages (TAM) in tumor microenvironment [47], and the up-regulated expression of immunosuppressive molecules for instance cytotoxic T lymphocyte associated antigen-4 (CTLA-4), also decreasing expression of cancer antigens which results the inactivation of tumor killing CD8 + T cells [56–59]. Therefore, we explored the probable relation between the gene signature and immune suppressive mechanisms. And the results revealed that the difference expression of ICD related genes in high-risk and low-risk groups of patients which were categorized based on the constructed gene signature, as well as the statistical significant correlation between the signature and ESTIATE immune score supported the signature was immune modulation associated. Further, we investigated the expression of immune checkpoints including PD-L1, CTLA4, TIGIT, TIM-3 and LAG-3 between the high-risk and low-risk groups of patients, and the results showed the high-risk patients had higher expression of CTLA4, LAG3 and TIGIT than the low-risk patients indicating the immune targeting potential for this group of ccRCC patients.

As the results of the relation analysis between the signature and 22 TICs indicated that the signature was significantly related with CD4( +) T memory cells and resting mast cells infiltration, and not only the two immune cells were related with the signature, but also they were able to predict ccRCC patients prognosis. CD4( +) T memory cells have already been reported to confer vital functions on malignancy immune regulation, including participating in the activation of CD8 + T and NK killing cells, involving in the tumour immunological reactions [60, 61]. And mast cells were reported to be able to not only influence tumor expansion via inducing angiogenesis and changing tumor extracellular matrix composition, but also could influence the infiltration and activity of dendritic cells, tumor-associated macrophages and lymphocytes, promoting pro-inflammatory reactions in tumor microenvironment [47, 62, 63]. The association between the signature and immune checkpoints expression as well as different immune cells infiltration, suggesting the stronger immunosuppressive environment in high-risk groups of patients comparing to low-risk group, highlighting the potential of this group to benefit from further clinical immunotherapy.

## Conclusion

The present study defined a four genes containing signature based on ccRCC genes expression information, the signature was not only closely associated with patients survival, but also immune regulation related. Multiple in vitro experiments data analysis supported the association between signature and ccRCC microenvironment immune aspects including immune checkpoints expression and various types of immune cells infiltration. Although the current result is not yet enough to support the application of the signature in clinical medical immunotherapy, rigorous prospective studies performed on animal models as well as clinical trials are still needed, the results shall provide meaningful insight into better understanding of the disease and shed lighting on further ccRCC immune regulation researches.

## Supplementary Information

**Additional file 1:** **Supplementary Figure 1.** Genetic alterations of four hub genes based on cBioPortal dataset. Different types of (A) MMP9, (B) IRF7, (C) NFKB1 and (D) HMOX1 variations including gene amplification, deletion, mutation and structural variants in various human cancers revealed by cBioPortal dataset.

**Additional file 2:** **Supplementary Figure 2.** GEO profiles validating the prognosis correlation of the constructed gene signature. (A) GSE22541 patients were divided into high-risk and low-risk groups based on the calculated signature score. (B) The survival status of all the GSE22541 patients samples. (C) Survival analysis of the high-risk and low-risk groups of GSE22541 patients. (D) ROC curve of the gene signature to predict GSE22541 patients survival. (E) Relative expression of MMP9, IRF7, NFKB1 and HMOX1 genes in GSE22541 samples displayed in a heatmap.

**Additional file 3:** **Supplementary Table 1.** Detailed information of the GEO datasets used for identifying differently expressed genes in ccRCC vs normal renal tissues. **Supplementary Table 2.** Number of the four levels differently expressed genes in ccRCC analyzed based on four GEO profiles. **Supplementary Table 3.** Detailed information of the 269 differently expressed meanwhile immune modulation related genes in ccRCC.

## Data Availability

Publicly available datasets were analyzed in this study. The data can be found here: GSE53000:https://www.ncbi.nlm.nih.gov/geo/query/acc.cgi?acc=GSE53000. GSE53757:https://www.ncbi.nlm.nih.gov/geo/query/acc.cgi?acc=GSE53757. GSE68417:https://www.ncbi.nlm.nih.gov/geo/query/acc.cgi?acc=GSE68421. GSE71963:https://www.ncbi.nlm.nih.gov/geo/query/acc.cgi?acc=GSE71963. GSE22541:https://www.ncbi.nlm.nih.gov/geo/query/acc.cgi?acc=GSE22541. All data generated or analyzed based on the online datasets and other experiments during this study are included in this published article.

## Abbreviations

ccRCC: Clear cell renal cell carcinoma
GEO: Gene Expression Omnibus
PPI: Protein–protein interaction network
ICIs: Immune checkpoint inhibitors
TMB: Tumor mutation burden
ICD: Immunogenic cell death
TIC: Tumor infiltrating immune cells
TAM: Tumour-associated macrophages
