# Supplementary figures and images for: Immune regulation and prognosis indicating ability of a newly constructed multi-genes containing signature in clear cell renal cell carcinoma
Source: BMC Cancer. 2023 Jul 12;23:649. doi: 10.1186/s12885-023-11150-4 (PMC10337188; doi:10.1186/s12885-023-11150-4)

A

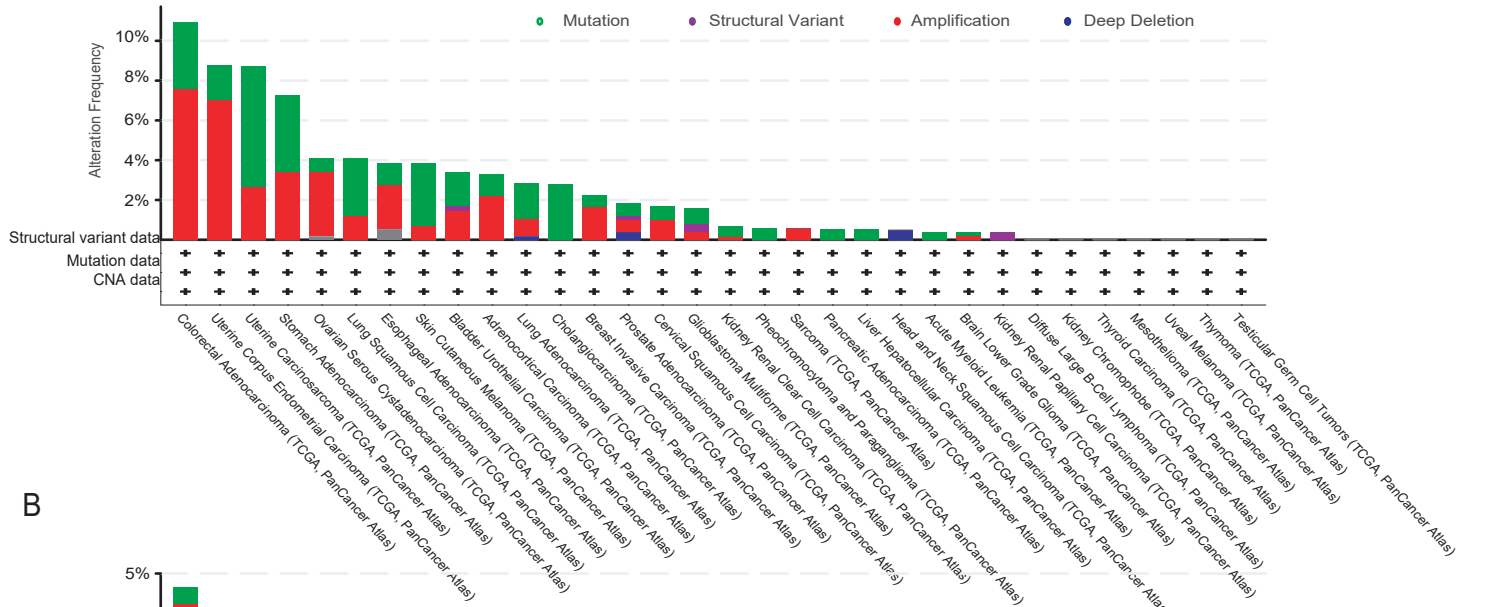

B

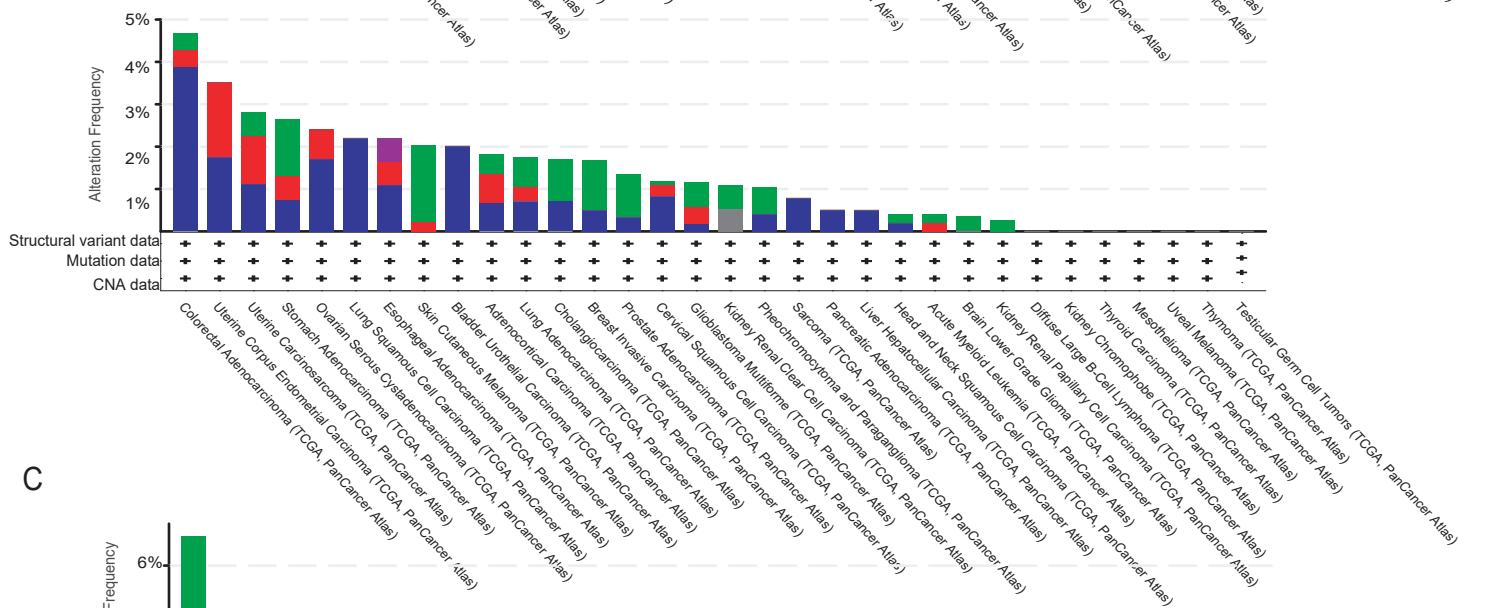

C

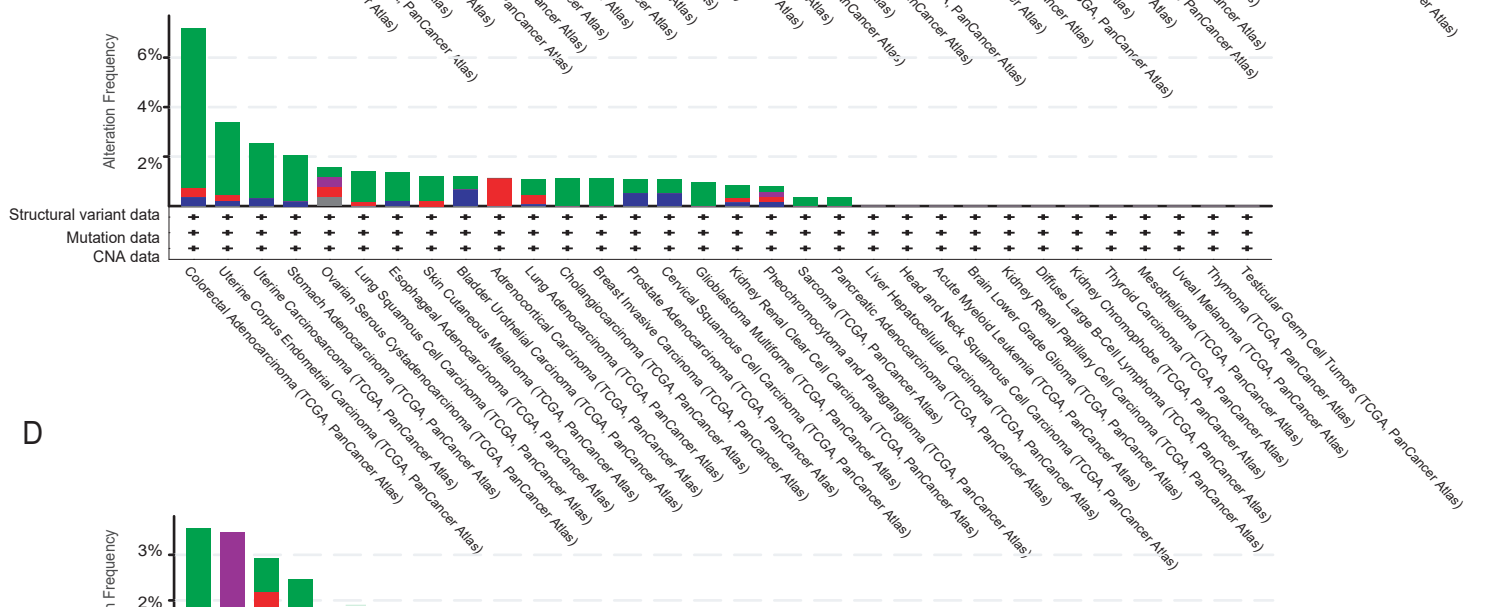

D

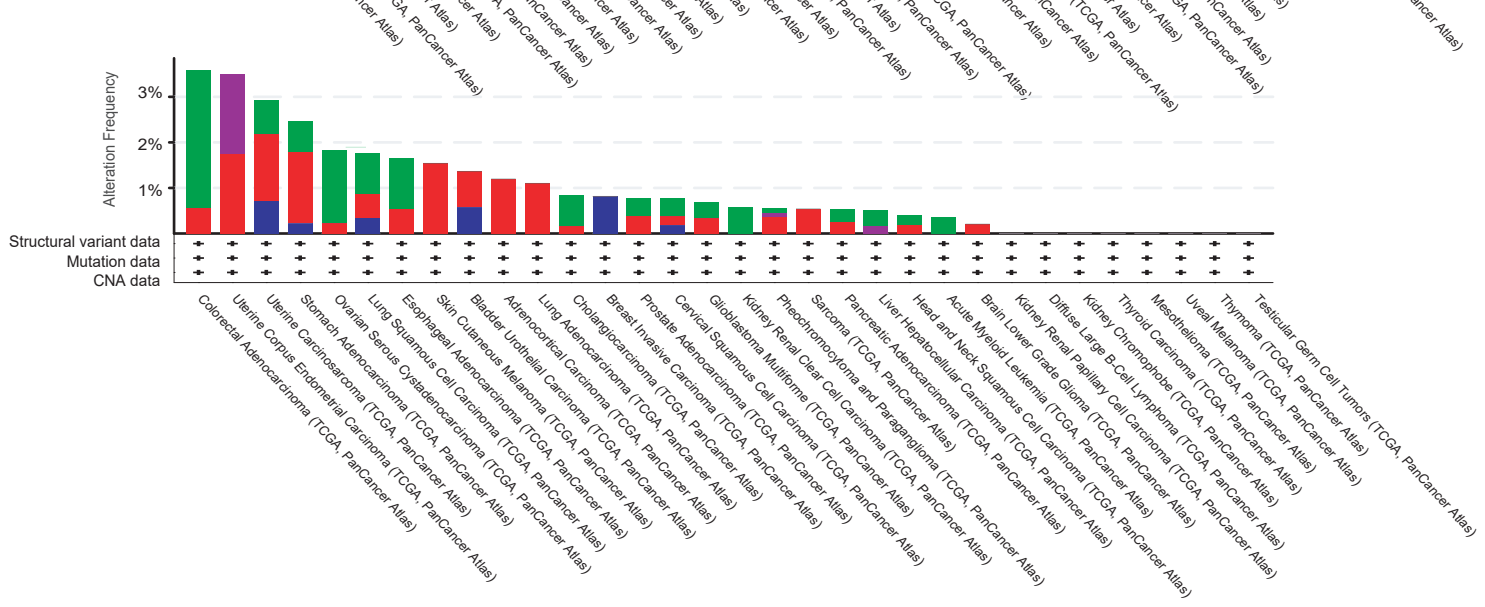

Supplement: Supplementary file 1 — Additional file 1: Supplementary Figure 1. Genetic alterations of four hub genes based on cBioPortal dataset. Different types of (A) MMP9, (B) IRF7, (C) NFKB1 and (D) HMOX1 variations including gene amplification, deletion, mutation and structural variants in various human cancers revealed by cBioPortal dataset. [file 12885_2023_11150_MOESM1_ESM.pdf]

A

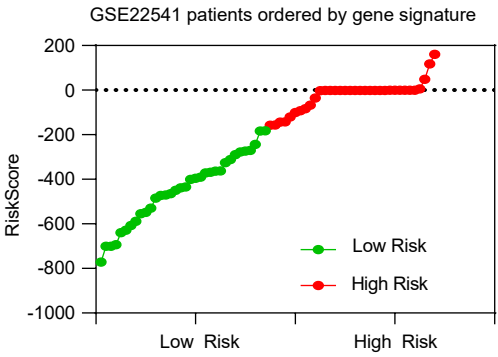

B

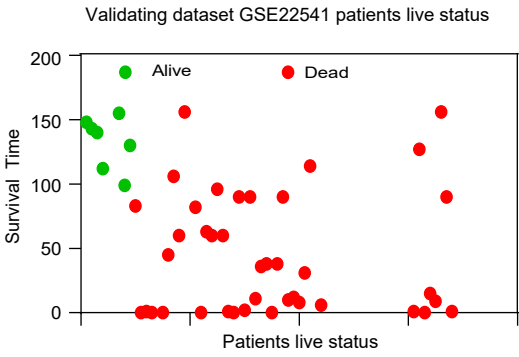

C

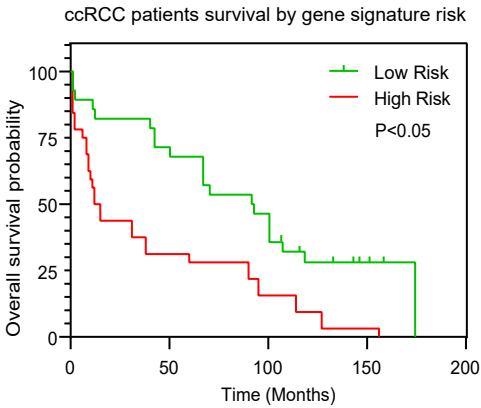

D

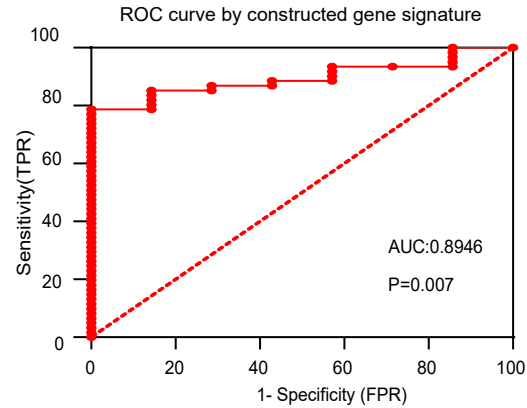

E

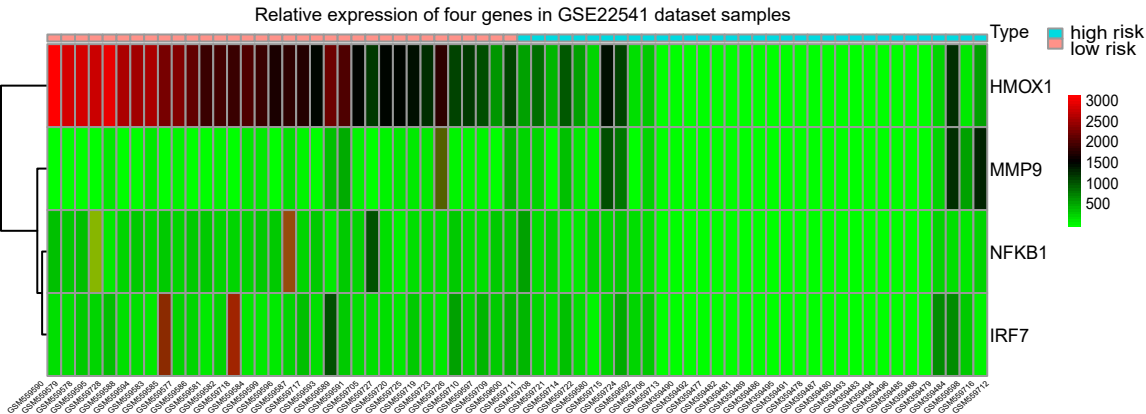

Supplement: Supplementary file 2 — Additional file 2: Supplementary Figure 2. GEO profiles validating the prognosis correlation of the constructed gene signature. (A) GSE22541 patients were divided into high-risk and low-risk groups based on the calculated signature score. (B) The survival status of all the GSE22541 patients samples. (C) Survival analysis of the high-risk and low-risk groups of GSE22541 patients. (D) ROC curve of the gene signature to predict GSE22541 patients survival. (E) Relative expression of MMP9, IRF7, NFKB1 and HMOX1 genes in GSE22541 samples displayed in a heatmap. [file 12885_2023_11150_MOESM2_ESM.pdf]
