# Supplementary material for: Immune regulation and prognosis indicating ability of a newly constructed multi-genes containing signature in clear cell renal cell carcinoma
Source: BMC Cancer. 2023 Jul 12;23:649. doi: 10.1186/s12885-023-11150-4 (PMC10337188; doi:10.1186/s12885-023-11150-4)
Supplement: Supplementary file 3 — Additional file 3: Supplementary Table 1. Detailed information of the GEO datasets used for identifying differently expressed genes in ccRCC vs normal renal tissues. Supplementary Table 2. Number of the four levels differently expressed genes in ccRCC analyzed based on four GEO profiles. Supplementary Table 3. Detailed information of the 269 differently expressed meanwhile immune modulation related genes in ccRCC. [file 12885_2023_11150_MOESM3_ESM.doc]

#### **Supplementary Table 1 Detailed information of the GEO datasets used for identifying differently expressed genes in ccRCC vs normal renal tissues**

| **GEO Datasets** | **Last Update** | **Contributor** | **Sample amount** | **Accessing website** |
| --- | --- | --- | --- | --- |
| GSE53000 | Jul 26, 2018 | Rowan A, Swanton C | 56 ccRCC and 6 normal renal samples | https://www.ncbi.nlm.nih.gov/geo/query/acc.cgi?acc=GSE53000 |
| GSE53757 | Mar 25, 2019 | von Roemeling CA, Copland JA.et al | 72 ccRCC and 72 normal renal samples | https://www.ncbi.nlm.nih.gov/geo/query/acc.cgi?acc=GSE53757 |
| GSE68417 | Jul 26, 2018 | Thibodeau BJ, Fulton M. et al | 29 ccRCC and 14 normal renal samples | https://www.ncbi.nlm.nih.gov/geo/query/acc.cgi?acc=GSE68417 |
| GSE71963 | Jan 23, 2019 | Takahashi M, Tsukamoto Y. et al | 32 ccRCC and 16 normal renal samples | https://www.ncbi.nlm.nih.gov/geo/query/acc.cgi?acc=GSE71963 |
| GSE22541 | Mar 25, 2019 | Wutting D, Fuessel S. et al | 68 primary and metastasis ccRCC samples | https://www.ncbi.nlm.nih.gov/geo/query/acc.cgi?acc=GSE22541 |

#### **Supplementary Table 2 Number of the four levels differently expressed genes in ccRCC analyzed based on four GEO profiles**

| **GEO datasets** | **<2 Fold** | **2~4 fold** | **4~8 fold** | **>8 fold** |
| --- | --- | --- | --- | --- |
| GSE53000 | 4270 | 1028 | 180 | 81 |
| GSE53757 | 21367 | 4857 | 1195 | 602 |
| GSE68417 | 8276 | 1425 | 290 | 159 |
| GSE71963 | 6266 | 3120 | 841 | 517 |

#### **Supplementary Table 3 Detailed information of the 269 differently expressed meanwhile immune modulation related genes in ccRCC**

| **269 genes that were indicated to be differently expressed in ccRCC meanwhile immune related** | | | | | | | |
| --- | --- | --- | --- | --- | --- | --- | --- |
| PDK1 | NDRG1 | C5AR1 | IL27RA | IL16 | CHP2 | FABP7 | LMBR1L |
| ANGPTL4 | PPARA | PTGER1 | CR2 | IDO1 | PSMD6 | LILRB3 | CD14 |
| KNG1 | GMFG | ACVR1B | CD70 | LYN | SEMA6D | IL15RA | CHP1 |
| HRG | TGFBR3 | TYROBP | PIK3R5 | GBP2 | TAP2 | BID | CYBB |
| PTH1R | CD40 | PROC | ESRRB | IFNGR2 | IFNGR1 | CD86 | HSPA2 |
| SEMA5B | TAP1 | ESRRG | FABP6 | TNFSF13B | SORT1 | NR3C2 | FGR |
| NOS1 | FGF1 | FAM3B | S100A10 | NRP2 | TYMP | PGF | THRB |
| PRLR | BPHL | IFNAR2 | PML | TLR2 | HLA-DPA1 | TAPBP | CXCL10 |
| ALB | DEFB1 | ITGB2 | PTPRC | TMSB10 | JAK1 | ANOS1 | LYZ |
| PSMB8 | CTSS | TKFC | OSMR | APOBEC3G | TNFRSF1B | BTK | SEMA3F |
| TGFA | NR0B2 | PLCG2 | JAG2 | TNFAIP3 | CANX | IL3RA | DCK |
| FGF9 | MSR1 | ESM1 | INPP5D | ICAM1 | ANGPTL2 | CYLD | STC2 |
| CXCR4 | NR1I3 | NCK1 | BMP7 | APOBEC3C | IL4R | HLA-DPB1 | PLXNC1 |
| ZC3HAV1L | ISG20 | VIM | TLR8 | CSF2RB | MAPK1 | RAC2 | FGF11 |
| VEGFA | CMTM4 | EGF | PRF1 | TRIM22 | HLA-DMB | CCL5 | NR5A2 |
| TLR3 | C3 | AZGP1 | TNFRSF10B | CAT | PIK3CG | ITGAL | TLR1 |
| ACKR3 | EGFR | NUDT6 | HLA-DMA | HCK | SHC1 | IL7 | VAV1 |
| ADM | PROCR | MET | GNLY | IL10RA | CHGB | RASGRP3 | ITK |
| WFDC2 | LCP2 | NR3C1 | TGFB1 | CD1D | TLR7 | CXCL16 | BMPR1B |
| CARD11 | FLT1 | VDR | PLXND1 | MICB | PLXNB1 | AEN | TNFSF8 |
| BLNK | SLIT2 | FCER1G | S100A2 | CRLF3 | KL | C3AR1 | CSF2RA |
| SSTR2 | CALCRL | FAS | EBI3 | DDX58 | IL32 | GCGR | ZYX |
| CBL | CMTM3 | CD72 | CXCL13 | FGFR2 | CD3G | FGFR3 | MMP9 |
| DLL4 | IL2RB | IL18RAP | SDC3 | TEK | IL1RAP | CSF1 | UNC93B1 |
| CSK | SLC11A1 | KLRD1 | PLAUR | LIMS1 | PAK5 | LTBP4 | PLXNA1 |
| BMP1 | CD4 | GRK2 | TAC1 | APLN | MTNR1A | TNFSF4 | PTGDR |
| IFIH1 | FCGR2B | NCK2 | KITLG | CD3E | NR2F1 | PAK2 | RAC1 |
| LRP1 | PDGFRA | PTGER3 | TNFRSF9 | SEMA3G | NFKB1 | BTC | SEMA6A |
| SH3BP2 | NOD1 | HSP90AA1 | TNFRSF1A | AQP9 | NR1H3 | NMB | NFATC1 |
| NRG3 | TNFRSF10A | CSF3R | PLXNA2 | ERAP2 | RAET1E | ERAP1 | B2M |
| PDGFD | IL1RL1 | HMOX1 | CXCL12 | SOS1 | NEDD4 | ANGPTL1 | OAS1 |
| IL12RB1 | TNFSF15 | PTGDS | PRDX2 | BACH2 | APOBEC3F | TNFSF9 | SEMA4D |
| CCL18 | PTHLH | PTPN6 | SOD1 | PTGFR | SEMA3B | CBLB | CBLC |
| IL17RA | IRF7 | CD48 | CSF1R | NFKBIE |  |  |  |

#### 
